# Two species of *Caiusa* Surcouf (Diptera: Calliphoridae) new to India, with data on larval behaviour and morphology

**DOI:** 10.3897/BDJ.6.e27736

**Published:** 2018-09-10

**Authors:** Ananda Banerjee, Knut Rognes, Daniel Whitmore

**Affiliations:** 1 BC Roy Road, nr Shyamkhola More, PO South Jagaddal, Rajpur, South 24 Parganas, Kolkata, West Bengal, India BC Roy Road, nr Shyamkhola More, PO South Jagaddal, Rajpur, South 24 Parganas Kolkata, West Bengal India; 2 University of Stavanger, Stavanger, Norway University of Stavanger Stavanger Norway; 3 Natural History Museum, London, United Kingdom Natural History Museum London United Kingdom

**Keywords:** blow flies, Oriental Region, Phumosiinae, Rhacophoridae, tree frogs, West Bengal

## Abstract

*Caiusa* Surcouf (Diptera: Calliphoridae) is an Old World genus of blow flies, the larvae of which feed on egg masses in the foam nests of various species of rhacophorid tree frogs. Here, we provide the first records for India (West Bengal, Eastern India) of *Caiusa coomani* Séguy, 1948 and *C. karrakerae* Rognes, 2015, together with new information on the behaviour and morphology of their larvae. Active surface swimming to disperse from infested nests is documented in blow fly larvae for the first time, as is the presence of a large internal air sac presumably acting as a floating aid. *Chiromantis simus* (Annandale, 1915) (Anura: Rhacophoridae) egg masses are first recorded as a feeding substrate of *Caiusa* larvae.

## Introduction

The blow fly genus *Caiusa* Surcouf (Diptera: Calliphoridae: Phumosiinae) was recently revised and contains eight valid species described to date, distributed in the Oriental, Australasian and Oceanian regions (Rognes 2015). The larvae of seven species have been bred from egg masses of one or more species of tree frog in the genera *Chiromantis* Peters, *Feihyla* Frost et al., *Polypedates* Tschudi and *Rhacophorus* Kuhl & Hasselt (Anura: Rhacophoridae), and appear to be key contributors to embryo mortality in these frogs (Lin and Lue 2000).

Prior to this study, two species of *Caiusa* were known to occur in India: the type species *C. indica* Surcouf, 1920 and *C. testacea* Senior-White, 1923, both recorded from South India (Surcouf 1920; Senior-White et al. 1940; Rognes 2015). The aim of this paper is to publish the results of a study carried out by the first author in West Bengal (Eastern India) between 2007 and 2018, which led to the discovery of two new species of *Caiusa* for the Indian fauna, *C. coomani* Séguy, 1948 and *C. karrakerae* Rognes, 2015, of new behavioural and morphological information on mature larvae of *Caiusa* species, and of a new association between *Caiusa* and the tree frog *Chiromantis simus* (Annandale, 1915) (Anura: Rhacophoridae).

## Materials and methods

**Figure 2.**
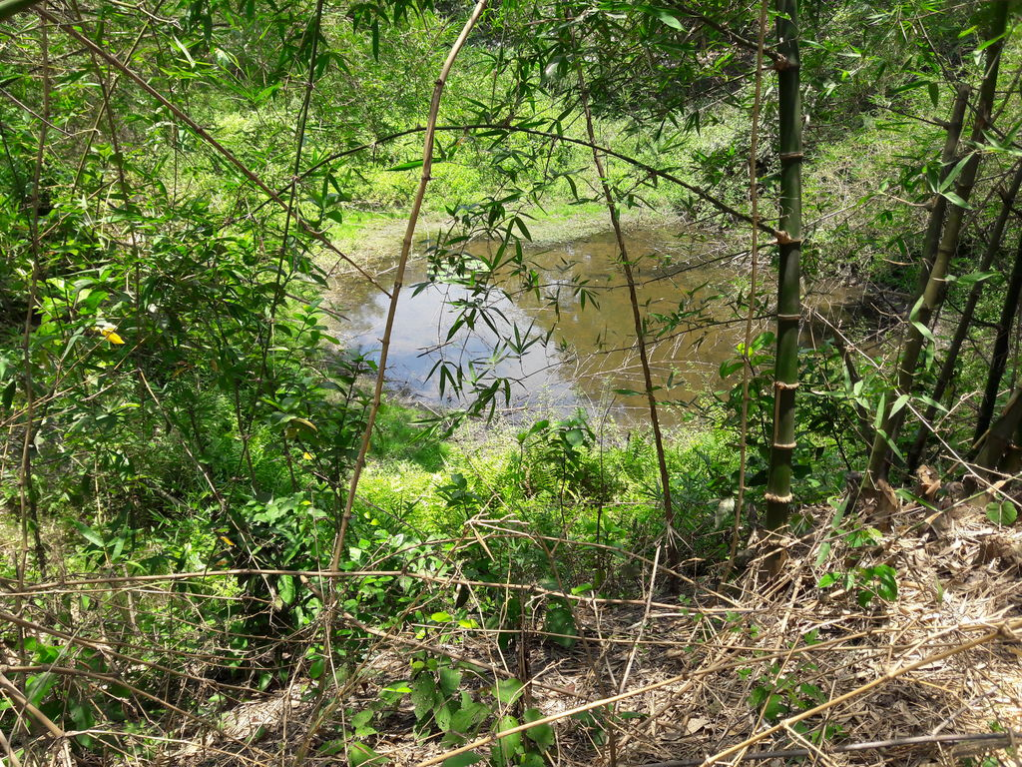
A small pond at Shyamkhola More (West Bengal, India).

**Study area.** Observations of live adults and larvae of *Caiusa* and rearings of adult flies from the foam nests (containing egg masses) of two tree frog species, *Chiromantis simus* (*Fig. 1a*) and *Polypedates leucomystax* (Gravenhorst, 1927) (Fig. 1b) (Anura: Rhacophoridae), took place between 2007 and 2018 at the residence of the first author near Shyamkhola More, 22°42′36″N 88°39′23″E (Eastern India, West Bengal, Rajpur Municipality) (Fig. 2).

**Frog biology and nest rearing.** Many frogs in the family Rhacophoridae lay egg masses in foam nests over puddles and abandoned ponds (Fig. 2) during the reproductive season (Rognes 2015). At Shyamkhola More, *Chiromantis simus* breeds during the monsoon period between June and October (Banerjee 2010, Banerjee 2014) and builds 50–100 foam nests per waterbody per year, each measuring about 6.0 cm x 3.0 cm x 2.5 cm (Deuti 2001, Banerjee 2014). *Polypedates leucomystax* is a larger species; at Shyamkhola More, it builds one to two foam nests (about 6.5 cm x 6.0 cm x 7.0 cm) per year, during the hottest summer months (May–July) (Deuti and Banerjee 2005, Banerjee and Deuti 2006). Infested foam nests of *Chiromantis simus*, hanging from leaves or twigs, were collected (about four to five per year) and kept individually in glass jars covered with a fine cloth for ventilation. The jars were kept indoors and cleaned every day to remove the liquid discharged from the nests. *Caiusa* larvae pupariated at the bottom of the jars under the leaves to which the foam nests were attached.

**Preservation and identification of *Caiusa* specimens.** Adult *Caiusa* specimens reared in captivity (Fig. 3) from frog nests were placed in air-tight containers with silica gel, killed in a freezer at sub-zero temperatures and pinned for long-term preservation. The abdomens of a small number of specimens were removed for dissection. Three males were sent to the second author for identification, using the key in Rognes 2015. All specimens preserved and dissected for this study are in the first author's private collection.

**Images and videos.** Figures 1, 3, 4b, 5, 6a and 7 were captured using a Panasonic Lumix FZ50 camera. Figures 2, 6b, 8, 9 and 10 were captured using a Samsung On8 smartphone. Figure 4a was captured with an Olympus E-420 digital camera with a 10 Mpx MOS sensor (17.3 x 13 mm), mounted by means of an LM-Scope photo adapter on a Wild M8 stereomicroscope equipped with a phototube (38 mm inner diameter).

## New records

The adult flies reared from *Chiromantis simus* egg masses at Shyamkhola More were identified as belonging to two species: *Caiusa coomani* (Fig. 4a) and *C. karrakerae* (Fig. 4b), both of which are here newly recorded from India; only *C. karrakerae* was identified among larvae feeding on *Polypedates leucomystax* egg masses in the study area, but only one specimen emerged from nests of this frog was dissected and identified to species. *Caiusa coomani* was previously known from Hong Kong, Malaysia, Singapore, Thailand and Vietnam (Rognes 2015), whereas *C. karrakerae* was recently described from Malaysia and Thailand. Four species of *Caiusa* are currently known to occur in India: *C. coomani*, *C. indica*, *C. karrakerae* and *C. testacea*. Those reported here are the first records of predation by *Caiusa* species on egg masses of the tree frog *Chiromantis simus*.

## Biology, larval behaviour and larval morphology

**Oviposition on *Chiromantis simus* nests.** Adult females of *Caiusa* sp. were seen ovipositing on *Chiromantis simus* nests at Shyamkhola More, and a maximum of three specimens were observed at any given time (Fig. 5a). Lin et al. (2000) and Lue and Lin (2000) studied the oviposition behaviour of *Caiusa violacea* Séguy, 1925 (as *C. coomani*) and suggested that the flies lay their eggs in the few hours after the foam nests are formed, when the outer surface of the foam is still soft. At Shyamkhola More, oviposition was observed between 5 and 8 am, about seven hours after construction of the foam nests; the flies never visited the foam nests later in the day. In *Chiromantis simus*, tadpoles drop from the foam nest on the third day (Deuti 2001). This suggests that, possibly, oviposition took place early in the morning not only to avoid drying and hardening of the outer surface of the foam nest but also to ensure maximum time for development of the larvae. It can be hypothesized that the frog eggs are easier to prey upon by *Caiusa* larvae than the more mobile tadpoles (Menin and Giaretta 2003).

**Abundance of *Caiusa* larvae in *C. simus* and *P. leucomystax* nests.** The number of *Caiusa* larvae found in infested *Chiromantis simus* foam nests varied between one and eight, most commonly six to seven. Besides the eggs, the larvae generally consumed the entire nest before pupariation, including the protective foam (Fig. 5b); in indoor conditions, pupariation took place at the bottom of the glass jars used to contain the nests. During this study, two infested nests of *Polypedates leucomystax* were observed, without being removed from their habitat. In July 2017, a foam nest of *P. leucomystax* containing at least 47 *Caiusa* larvae was seen after it had dropped into the water about one foot away from the margin. After one day, the nest had drifted towards the margin and the fly larvae crawled onto land and hid in cracks in the soil and under bricks, earthen tubs and plastic buckets to pupariate; inspection of the nest showed that most of the frog's eggs had been consumed but that the larvae had not eaten the foam. A mosquito net was erected above the area where the fly larvae had settled to pupariate. After five to six days, 27 *Caiusa* adults emerged, one of which was identified as *C. karrakerae*; the rest of the larvae either did not produce adults or had dispersed beyond the mosquito net. Similarly, in June 2018, 19 *Caiusa* larvae were observed while dispersing from a fallen *P. leucomystax* nest (Figs 6b, 8).

**Behaviour of third instar *Caiusa* larvae.** Because the foam nests of rhacophorid frogs are usually hanging from vegetation above the water, the question of how mature *Caiusa* larvae reach dry land to pupariate has remained open. Observations of *C. simus* and *P. leucomystax* nests during this study have provided new information on the dispersal of mature *Caiusa* larvae from their feeding sites. At Shyamkhola More in September 2011, one larva was observed at night while descending from an outlier *C. simus* nest (suspended about 1 m above land at a short distance from the pond) by using a thread of unknown nature seemingly secreted by its posterior end (Fig. 6a). A bucket of water was placed below the nest to intercept the larva. After reaching the water, the larva swam to the edge of the bucket (Fig. 7) and slowly climbed out to find a suitable place to pupariate. In early June 2018 a similar behaviour was observed by *Caiusa* larvae dispersing from a *P. leucomystax* nest floating on the water (Fig. 6b); one larva was placed in a bucket and swam for over five hours (Fig. 8). The efficient swimming mechanism documented in the videos in Figs 7, 8 suggests that this is a habitual behaviour of *Caiusa* larvae; together with the thread used to reach the water surface, it provides a plausible explanation of how larvae of these blow flies reach dry land to pupariate after feeding in foam nests suspended above the water. To our knowledge, this type of swimming behaviour had never been observed in a blow fly larva before. The swimming ability and relative morphological adaptations (see below) of the larvae are possibly a new synapomorphy of species in this genus of frog predators, but we have no data on the other *Caiusa* species at present.

**Morphology of third instar *Caiusa* larvae.** The swimming behaviour observed in *Caiusa* larvae would appear to be enabled by at least one conspicuous morphological adaptation, in the form of a large internal air sac located in the abdominal segments of the larva (Fig. 9a) – also visible in Fig. 6b – and presumably acting as a floating aid. Dissection of a larva (Fig. 9b, c) showed that the air sac can be separated from the rest of the body without it deflating, which suggests the presence of a valve-like mechanism at the mouth of the sac (Fig. 9d). More detailed anatomical studies are needed to assess whether the possible inlet tube connected to the sac (Fig. 9d) is part of the tracheal system or part of the digestive system as has been documented in some aquatic Sciomyzidae larvae, which swallow air through their mouthparts for more efficient swimming (see Rozkožný 1984). Our observations show that *Caiusa* larvae curl their posterior end toward the water surface during brief pauses in their swimming motion, presumably for air intake through the posterior spiracles (Fig. 10).

## Figures and Tables

**Figure 1a.**
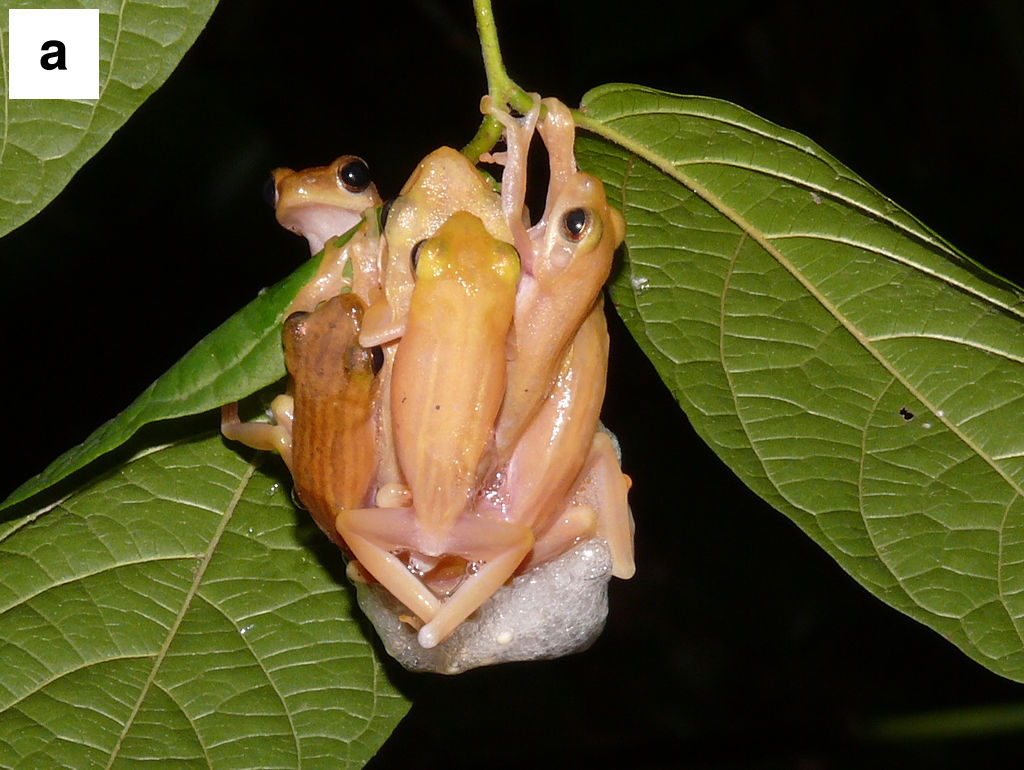
Amplexus of five males and one female of *Chiromantis simus* (Annandale).

**Figure 1b.**
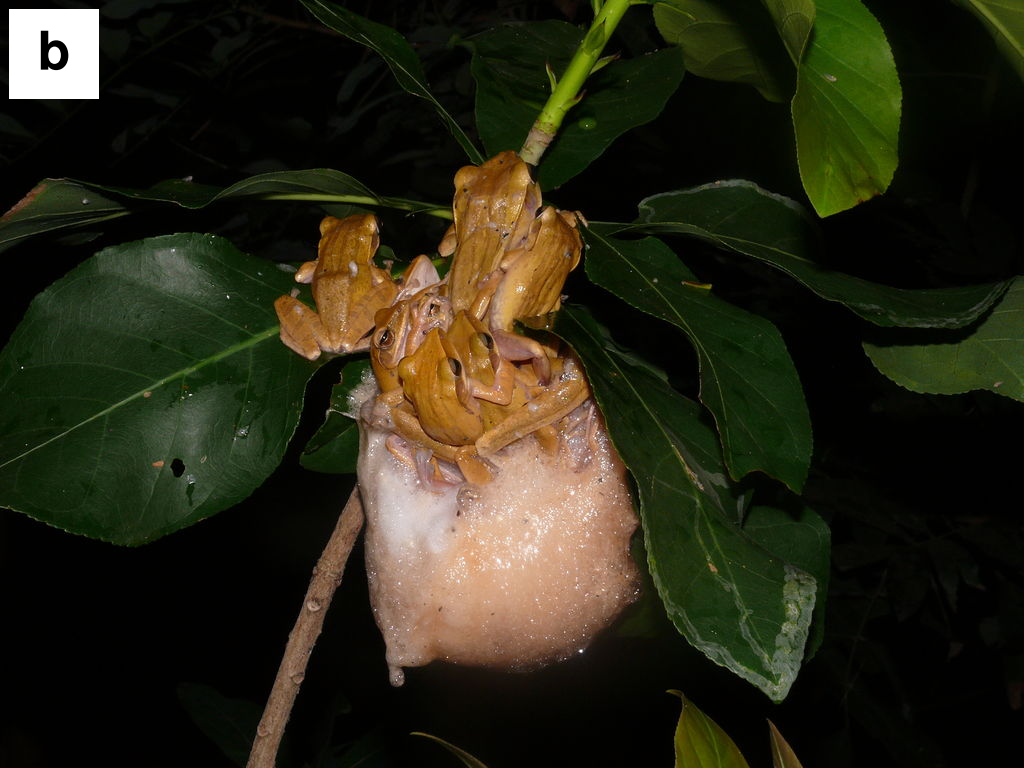
Amplexus of seven males and one female of *Polypedates leucomystax* (Gravenhorst).

**Figure 3a.**
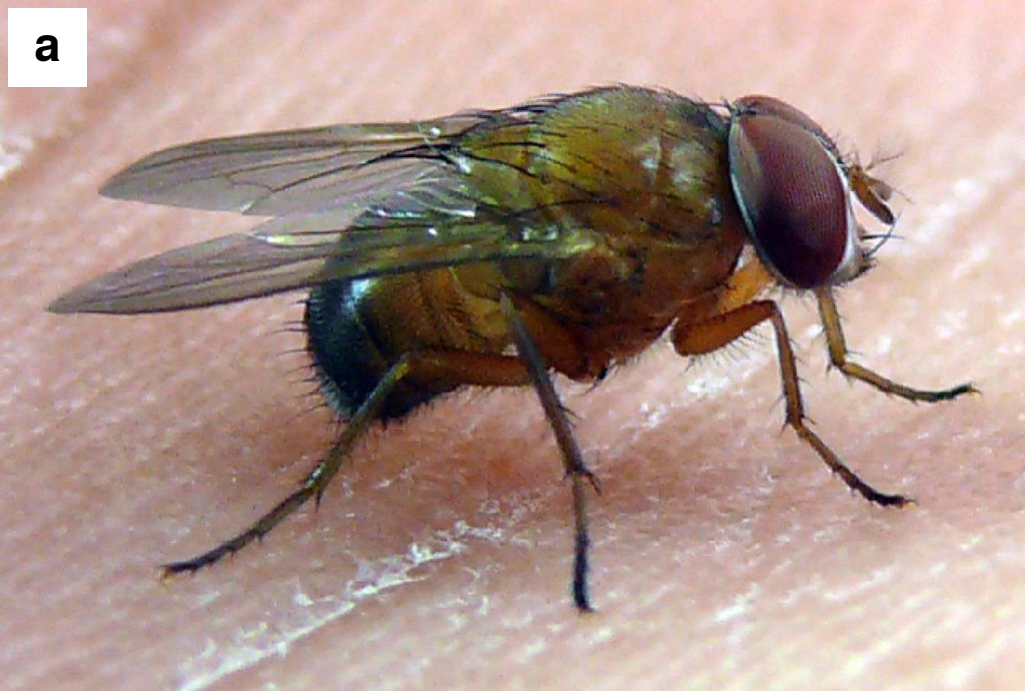
A live, newly emerged *Caiusa* sp. male resting on the palm of the first author's hand.

**Figure 3b.**
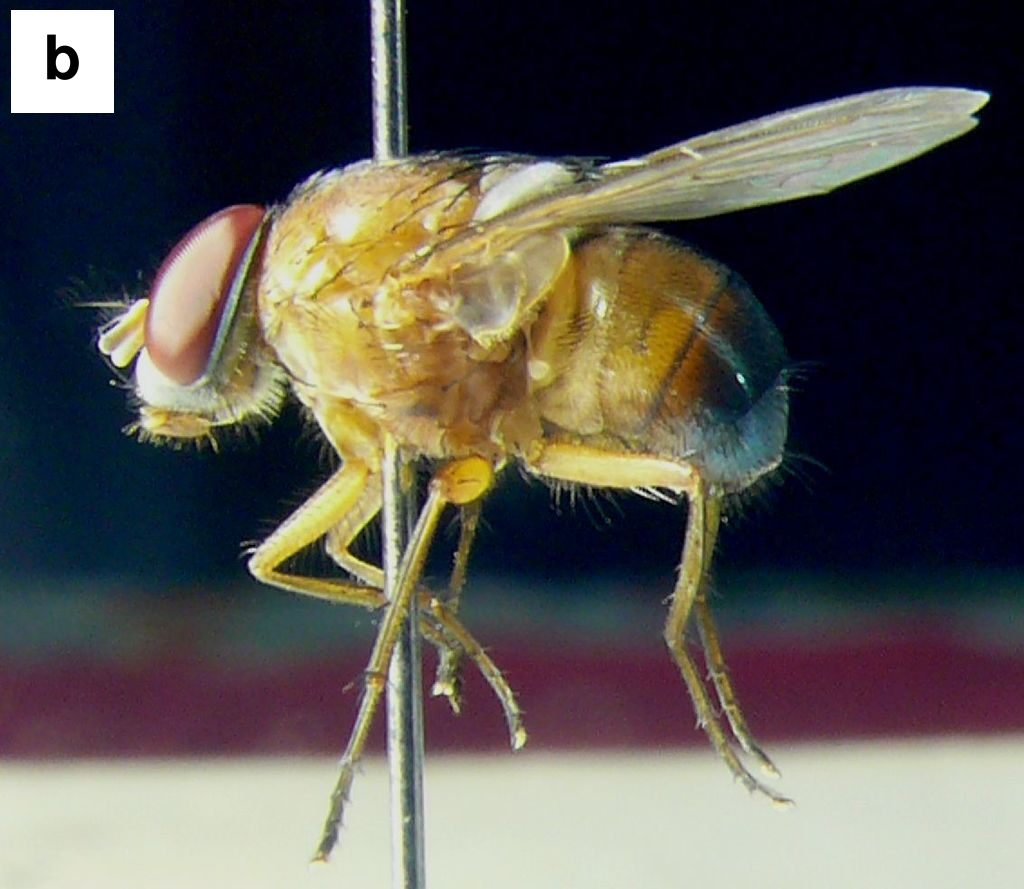
A pinned *Caiusa karrakerae* Rognes male.

**Figure 4a.**
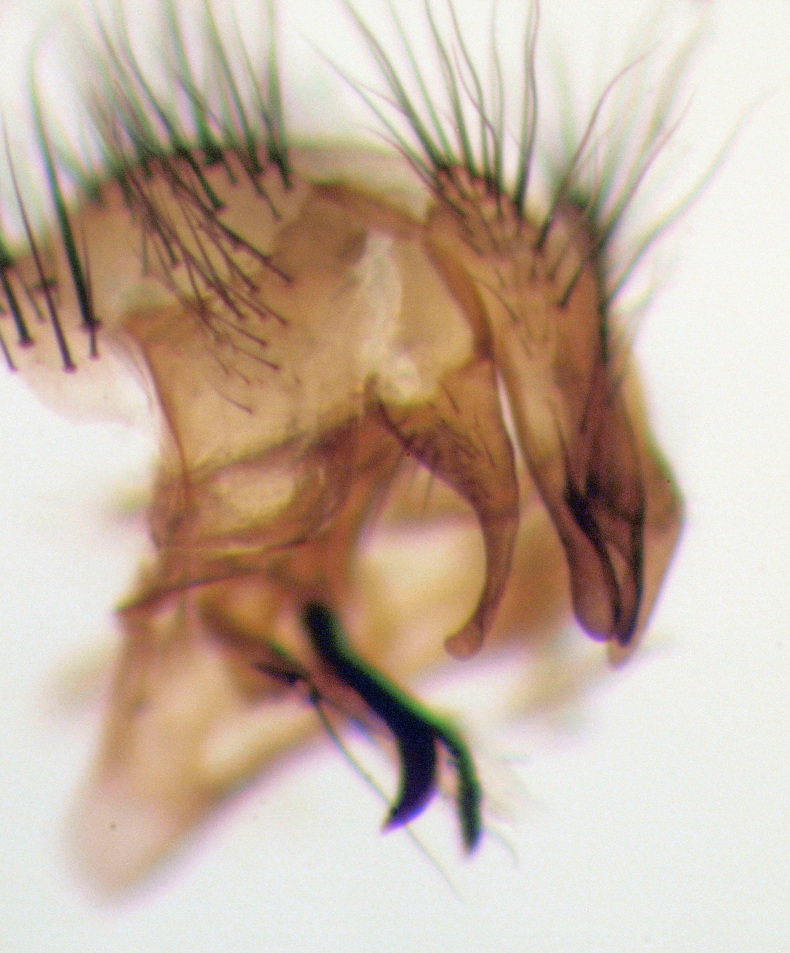
*Caiusa coomani* Séguy, 1948.

**Figure 4b.**
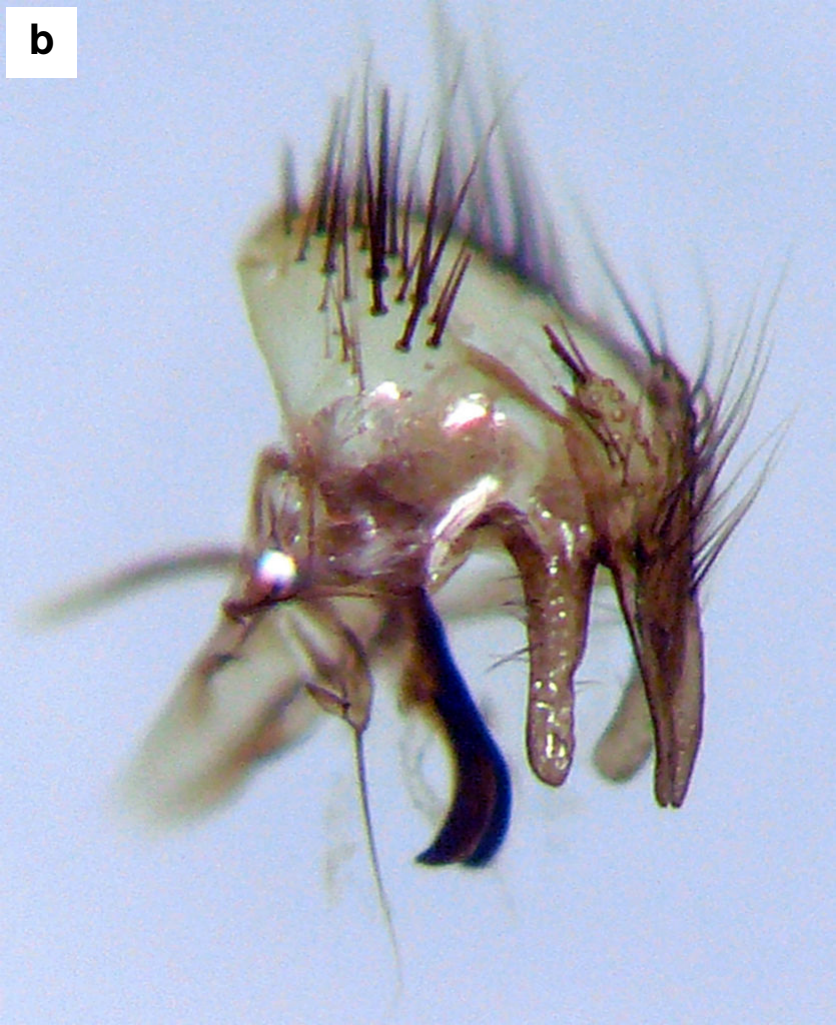
*Caiusa karrakerae* Rognes, 2015.

**Figure 5a.**
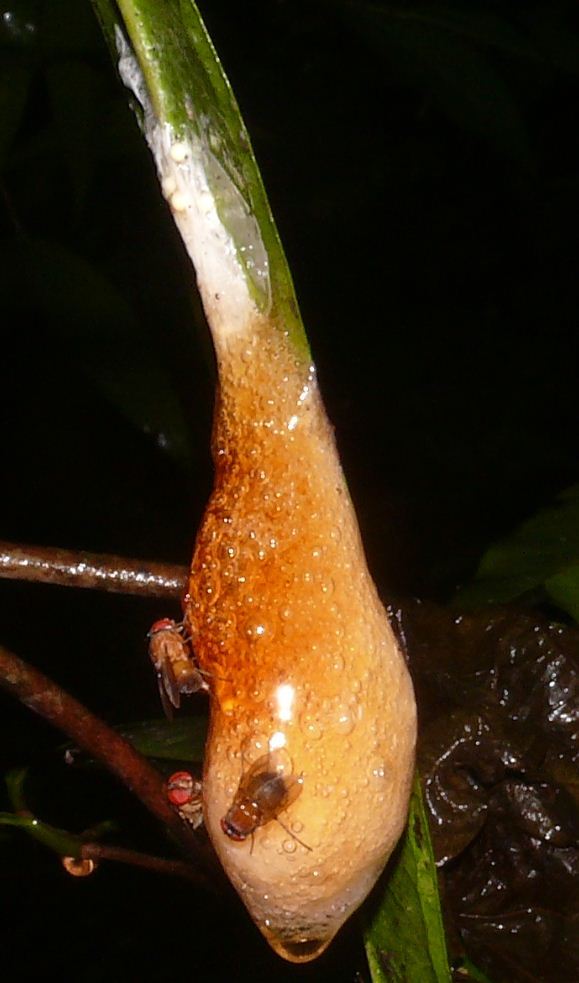
Three *Caiusa* females on the surface of a nest. The upper left female seems to be ovipositing.

**Figure 5b.**
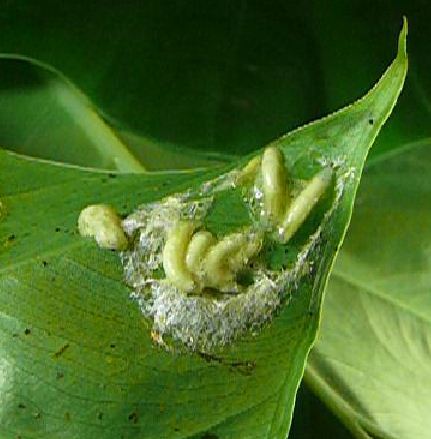
Remains of an infested nest containing seven *Caiusa* larvae. All the frog's eggs and foam were consumed by the larvae.

**Figure 6a.**
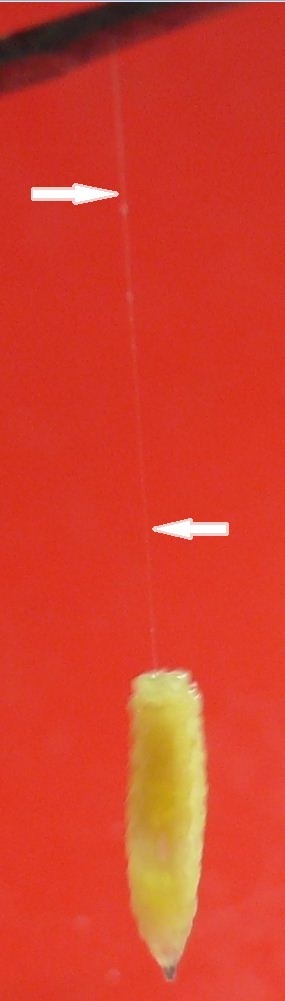
Larva descending from a *Chiromantis simus* (Annandale) nest using a thin thread (arrows).

**Figure 6b.**
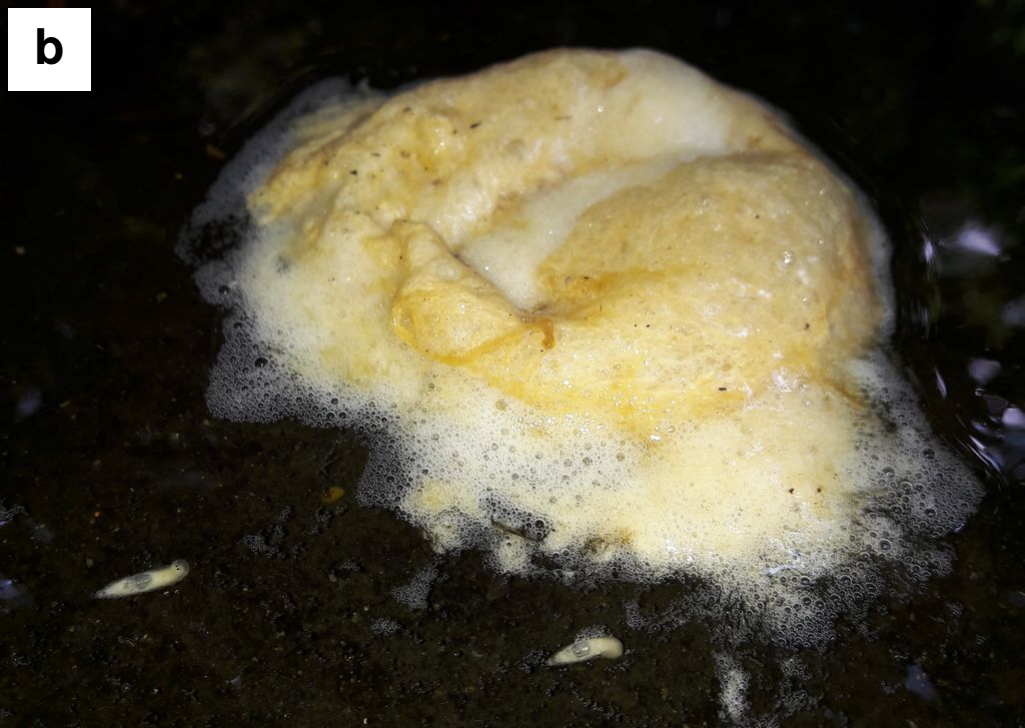
Larvae dispersing from a *Polypedates leucomystax* (Gravenhorst) nest.

**Figure 7.** Third instar *Caiusa* larva swimming in a bucket after it descended from a *Chiromantis simus* (Annandale) nest by means of a thread (Shyamkhola More, West Bengal).

**Figure 8.** Third instar *Caiusa* larva swimming in a bucket. It was collected after dispersal from a *Polypedates leucomystax* (Gravenhorst) nest at Shyamkhola More (West Bengal) and swam for over five hours.

**Figure 9a.**
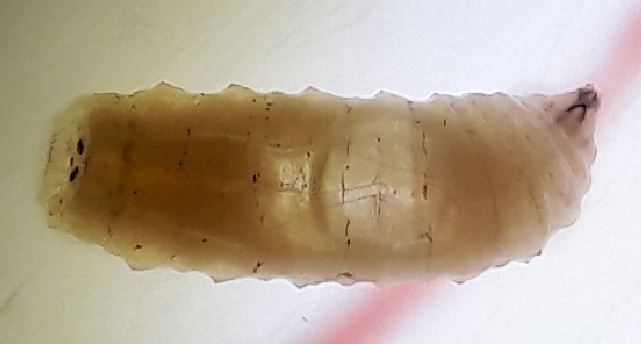
Habitus image showing air sac and tracheae.

**Figure 9b.**
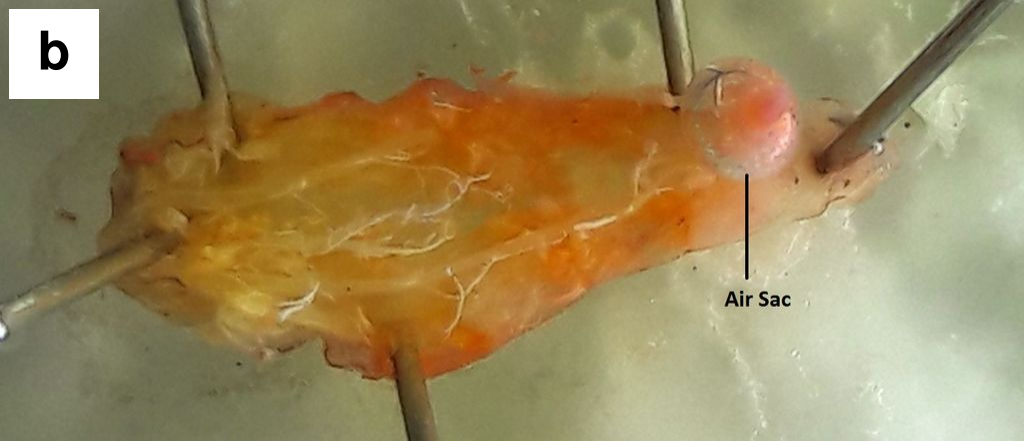
Dissected larva showing air sac.

**Figure 9c.**
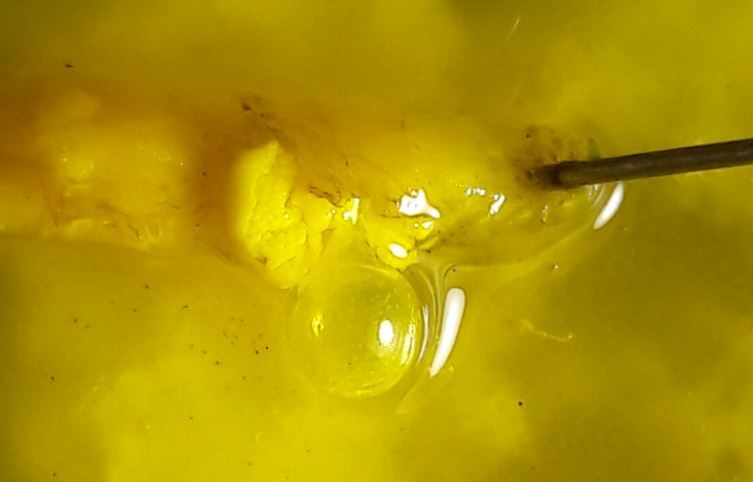
Air sac being separated from rest of larva.

**Figure 9d.**
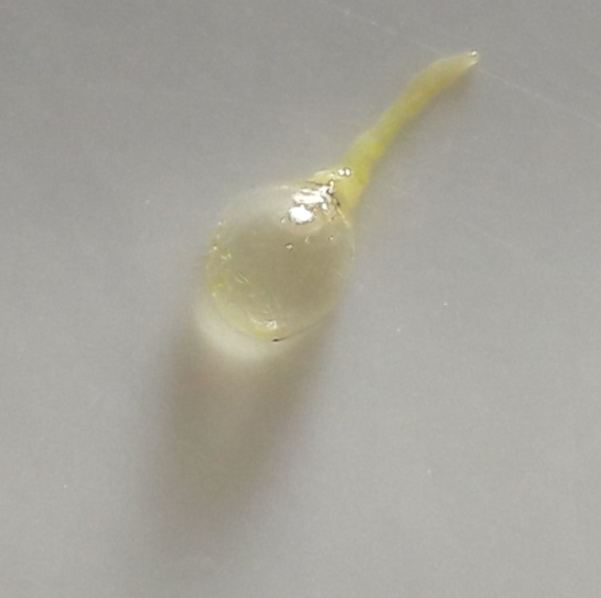
Dissected air sac and possible air inlet tube.

**Figure 10.** Third instar *Caiusa* larva collected from a *Chiromantis simus* (Annandale) nest at Shyamkhola More (West Bengal) in 2018. The video shows the larva pausing its habitual swimming motion, presumably for air intake through the posterior spiracles.
